# A Regression-Based Differential Expression Detection Algorithm for Microarray Studies with Ultra-Low Sample Size

**DOI:** 10.1371/journal.pone.0118198

**Published:** 2015-03-04

**Authors:** Daniel Vasiliu, Samuel Clamons, Molly McDonough, Brian Rabe, Margaret Saha

**Affiliations:** 1 Department of Mathematics, College of William and Mary, Williamsburg, Virginia, United States of America; 2 Department of Biology, College of William and Mary, Williamsburg, Virginia, United States of America; University of Jaén, SPAIN

## Abstract

Global gene expression analysis using microarrays and, more recently, RNA-seq, has allowed investigators to understand biological processes at a system level. However, the identification of differentially expressed genes in experiments with small sample size, high dimensionality, and high variance remains challenging, limiting the usability of these tens of thousands of publicly available, and possibly many more unpublished, gene expression datasets. We propose a novel variable selection algorithm for ultra-low-*n* microarray studies using generalized linear model-based variable selection with a penalized binomial regression algorithm called penalized Euclidean distance (PED). Our method uses PED to build a classifier on the experimental data to rank genes by importance. In place of cross-validation, which is required by most similar methods but not reliable for experiments with small sample size, we use a simulation-based approach to additively build a list of differentially expressed genes from the rank-ordered list. Our simulation-based approach maintains a low false discovery rate while maximizing the number of differentially expressed genes identified, a feature critical for downstream pathway analysis. We apply our method to microarray data from an experiment perturbing the Notch signaling pathway in *Xenopus laevis* embryos. This dataset was chosen because it showed very little differential expression according to limma, a powerful and widely-used method for microarray analysis. Our method was able to detect a significant number of differentially expressed genes in this dataset and suggest future directions for investigation. Our method is easily adaptable for analysis of data from RNA-seq and other global expression experiments with low sample size and high dimensionality.

## Introduction

Gene expression analysis has led to profound advances in our understanding of a wide array of biological processes ranging from ecology and evolution to molecular genetics and disease therapeutics (reviewed in [1, 2], and references therein). Although improvements in sequencing technologies have resulted in an increasing number of RNA-seq transcriptomic experiments, the vast majority of global gene expression analyses studies in the literature have employed a microarray approach.

However, while the technology required to conduct microarray experiments has become relatively straightforward, data analysis remains challenging. Virtually every aspect of data analysis, from normalization to analysis of differential expression, remains a topic of ongoing discussion and often controversy in the literature [3] and [4]]. A particularly challenging data analysis problem arises from the very aspect that makes this technology so powerful, namely the large number of genes assayed on a chip, typically on the order of tens of thousands. The necessity of correcting for multiple hypothesis testing [5] often results in the lack of statistical significance for many experiments, particularly those with a few samples. This scenario, coined the “*p* ≫ *n*” dilemma in literature, complicates statistical analysis and potentially diminishes the value of the experiment [6, 7].

There is a widespread and intense interest in developing new analytical strategies to address the “*p* ≫ *n*” problem, for the following reasons. Firstly, while early microarray experiments focused on samples with large differences in a few genes, more recent findings stress that it is not large changes in a few genes, but rather small changes in many genes that will be important for understanding both complex diseases and the subtleties of biological processes. While current methodologies work well when the differences between experimental conditions are dramatic, such methods are not appropriate for detecting subtle, more biologically relevant changes. Secondly, the number of genes queried (i.e. dimensionality) continues to rise with the inclusion of splice variants and other forms of data provided by “next-generation” approaches such as RNA-seq, a technology for which our approach will be applicable. Thirdly, in many instances, sample size is inherently limiting. In many experiments, including those in conservation biology (endangered species) and medical research (rare tumor subtypes), the investigator cannot increase the *n*. Finally, thousands of microarray datasets are archived in publicly available databases; novel analytical approaches may reveal new findings. In fact, at the time of this writing, 16.6% of the ∼40,000 NCBI GEO Datasets of the type “gene expression by array” have an *n* ≤ 5 and 6.9% have *n* ≤ 3. The fraction of GEO Datasets of unrestricted type is even higher (24.1% have *n* ≤ 5 and 12.7% have *n* ≤ 3). Many more studies likely remain unpublished and unavailable due to lack of differential expression detectable by widely-used analysis techniques.

Existing algorithms for differential expression detection in cases of ultra-low *n* (2 to 5) have been compared and reviewed by Kooperberg et al. [8], Jeffery et al. [9], Murie et al. [10], Jeanmougin et al. [11], and Tan et al. [12]. Kooperberg et al. [8], Murie et al. [10], and Tan et al. [12] showed that differential expression detection using independent t-statistics has weak power for small-sample-size analysis. In every review in which limma, a popular empirical Bayes technique, was tested (only Tan et al. did not), limma performed better than or comparably to every other method. However, limma is not sufficient to detect differential expression in all cases.

In the last decade, penalized regression techniques (reviewed by Ma and Huang [13]), including Lasso, elastic net, and SCAD have played a significant role in the “small n and large p” quandary in the general statistics literature [14]. These techniques were first employed in biostatistics for classification problems [15]. Specifically, penalized regression approaches have been widely applied to cancer diagnosis [16–19] and patient outcome prediction [20–22], as well as analysis of SNP data [23, 19].

One important feature of penalized regression methods is that they are variable selectors as well as classifiers. Building a classifier with penalized regression involves assigning a weight to each gene, which determines how strongly that gene contributes to the classifier. Differentially expressed genes receive high weight, while genes that do not vary much between conditions are assigned low weights. By separating genes with low weight from those with high weight, penalized regression can identify differentially expressed genes. Unfortunately, although differentially expressed genes are expected to have high weight and insignificant genes are expected to have low weight after penalized regression, there is no *a priori* definition of *how* high a gene’s weight must be to be differentially expressed. Most applications of penalized regression to variable selection use some form of cross-validation to assess the impact of individual variables on the accuracy of a classifier (see Du et al. [24] for an example as applied to arthritis and colon cancer datasets, or [25] for an example using several cancer datasets). Cross-validation involves splitting a dataset into a training set and a validation set. Regression is performed on the training set to produce a classifier. The classifier is then applied to the validation set to measure the classifier’s accuracy. This process can be repeated for different values of critical parameters (for example, the number of genes used by the classifier), and cross-validation measures the effect of the change on classifier accuracy. Cross-validation works well when applied to cancer datasets, which typically involve between dozens and hundreds of samples. However, cross-validation is unstable or impossible with extremely small sample sizes, making it inappropriate for microarray studies with low *n* [26].

Clearly novel approaches for analyzing *p* ≫ *n* data would be useful for high-throughput gene expression analysis. Here we propose to address this need by developing, applying and refining a novel method for analysis of microarray data broadly usable by biologists. Our method is based on penalized Euclidean distance (PED), a penalized binomial regression approach which performs favorably compared to similar methods such as elastic net, Lasso, SIS, and ISIS [27]. Our approach uses a simulation-based tuning procedure that eliminates the need for cross-validation and maximizes the number of selections made while maintaining an arbitrarily low false discovery rate (FDR). We apply this model to a microarray dataset that examined how *Xenopus laevis* embryos respond over time to injection with constructs that alter the Notch signaling pathway [28]. This was a particularly suitable dataset given that it showed minimal statistical significance when analyzed with commonly used analysis packages, e.g. limma, yet the most differentially expressed (but not significantly differentially expressed) genes according to limma included a number of genes known from other research to be involved in the Notch signaling pathway.

## Materials and Methods

### Microarray Experiment

A colony of *Xenopus* *laevis* was maintained as previously described [29] with all protocols approved by the College of William and Mary Institutional Animal Care and Use Committee (IACUC-2013-11-21-9110-MSSAHA) in accordance with federal guidelines. Embryos were obtained and raised using standard, published procedures [30].

Embryos were unilaterally injected into one blastomere at the two cell stage with 1.5 ng of one of the following capped RNA constructs synthesized *in vitro*: a DNA Binding mutant of Suppressor of Hairless (DBM), a construct that suppresses Notch signaling [31]; the Notch Intracellular Domain (NICD), which activates the Notch signaling pathway [32]; or Green Fluorescent Protein (GFP) as a tracer and control for the injection procedure. The DBM and NICD constructs were kind gifts from Dr. Chris Kintner. Capped RNA was synthesized in vitro using mMessage Machine (Ambion) following the manufacturer’s protocol and purified using the Qiagen MinElute Cleanup Kit. Embryos were raised to either late neurula stage (st. 18), tailbud stage (st. 28), or swimming tadpole stage (st. 38). All staging is according to Nieuwkoop and Faber [33].

To obtain total RNA, 10 embryos from each stage and condition were homogenized in Tri Reagent (Molecular Research Center) and extracted with 1-bromo-3-chloropropane phase separation reagent according to the manufacturer’s protocol. RNA from the aqueous phase was purified using the Qiagen RNeasy Mini kit. Total RNA for each of the nine samples (embryos injected with the three constructs NICD, DBM, GFP with each harvested at three different stages) was sent to the Clemson University Genomics Institute for microarray analysis using the Affymetrix Xenopus laevis 2.0 GeneChip. Affymetrix protocols were followed with the exception that the *in vitro* transcription reaction was carried out for 16 hours.

### Initial Statistical Analysis

Raw microarray data was normalized and summarized using Robust Microarray Average (RMA) [34] as implemented in the Bioconductor package [35] in R. For our initial statistical analysis, we reviewed five studies testing multiple differential expression detection algorithms at extremely low sample size [8–12]. All of the sources that reviewed limma recommended it over other algorithms. We therefore determined differential expression using the limma package [36] for R by fitting a linear model to produce p-values based on a moderated t-statistic. The following comparisons were employed: stage 18 DBM versus stage 18 GFP; stage 18 GFP versus stage 18 NICD; stage 28 DBM versus stage 28 GFP; stage 28 GFP versus stage 28 NICD; stage 38 DBM versus stage 38 GFP; and stage 38 GFP versus stage 38 NICD. Benjamini-Hochberg (BH) correction [37] was set with a false discovery rate of 0.05 to correct for multiple hypothesis testing. When the analysis was repeated using Benjamini-Yekutieli correction [38], no genes were selected as differentially expressed. Benjamini-Yekutieli correction is more conservative than BH correction, but is more suitable than BH correction or correlated data.

### Overview of Variable Selection by PED

**Fig 1:**
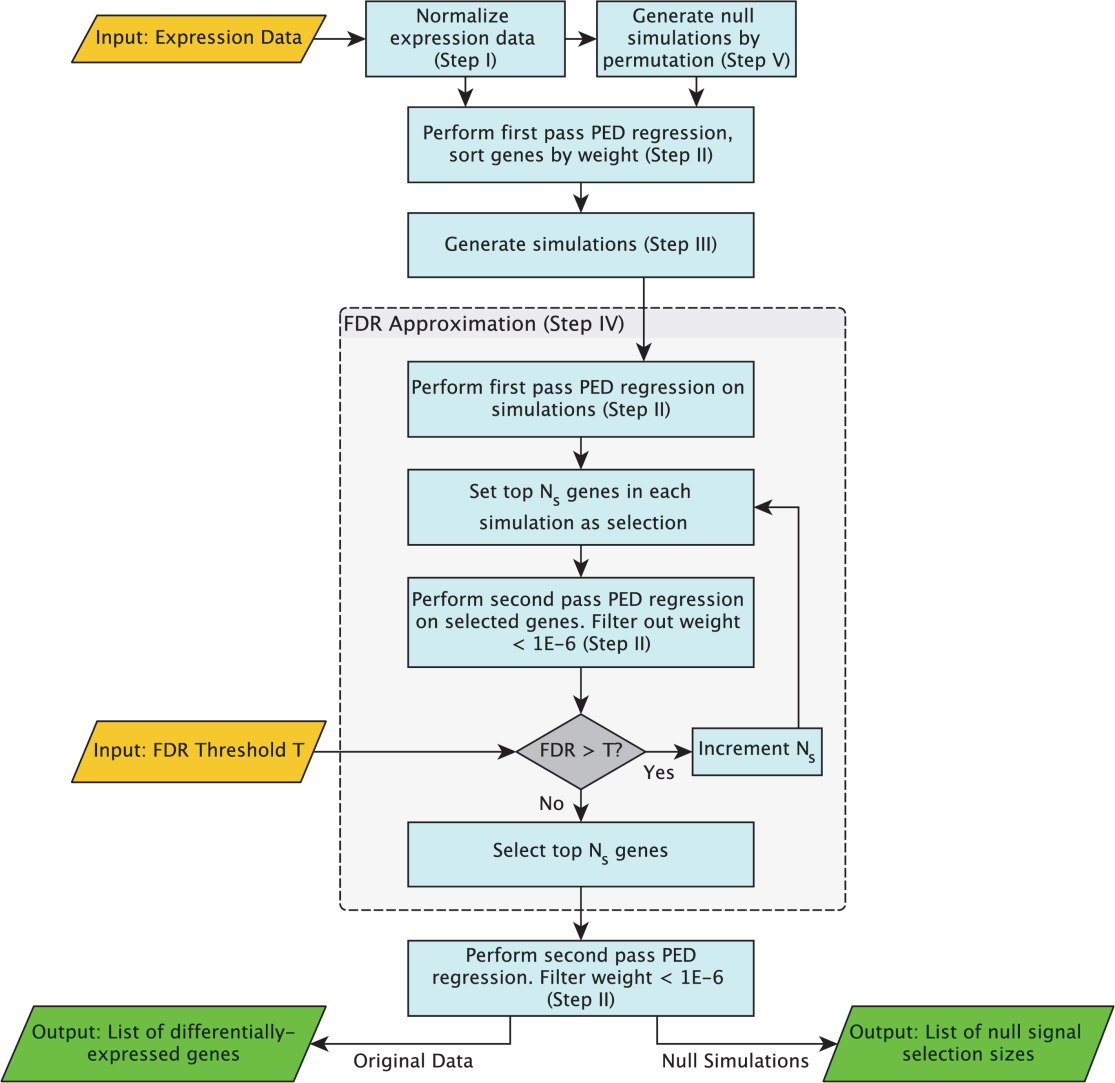
A schematic overview of gene selection by PED.

An overview of our selection method is shown in Fig. 1. First, we normalize the data by converting it to z-scores, so that each gene’s expression has mean 0 and standard deviation 1 (Step I). We then use PED regression, a form of penalized binomial regression, to rank the importance of each gene (Step II). PED regression produces a GLM-based classifier, which is a function that can identify the experimental condition of a microarray based on a linear combination of that microarray’s expression data. The classifier is defined by a vector of parameters, or weights, which are assigned to the set of genes in the experiment by PED. These weights determine the importance of each gene to the classifier—the higher the weight of a gene, the more information it contributes towards making a correct classification. Ideally, non-differentially expressed genes would have zero weight, and only differentially expressed genes would have non-zero weight. Due to both computational and signal-recovery limitations, in practice weights of non-differentially expressed genes can be quite small, but are rarely exactly zero. Thus, a challenge when using penalized regression methods for variable selection is to determine exactly which weights are large enough to indicate differential expression.

To determine a cutoff for significance, we generate simulations based on the experimental data (Step III). Starting from the most highly-ranked genes, we consider increasingly more genes to be provisionally differentially expressed, then use our simulations to estimate the false discovery rate of that selection. We increase the number of differentially expressed genes until the false discovery rate rises above a user-set threshold, at which point we stop and the selection is reported (Step IV). Finally, permutations of the original data, which contain the same data but with experimental labels scrambled, are analyzed as a null-signal control to test for overall presence of differential expression in the dataset (Step V).

Descriptions of each step of the method and several important implementation details are presented below.

#### Step I: Normalization

The average expression level of two different genes can easily differ by several orders of magnitude. Differences in the scales of gene expression can bias the results of penalized regression, which we use to rank the importance of genes. To prevent this bias, raw expression data are first centered and normalized by converting them into z-scores (so that each gene has average expression 0 and standard deviation of expression 1).

#### Step II: PED Regression

Our algorithm ranks the estimated importance of genes using PED, with a generalized linear model-based method. Generalized linear models are powerful and flexible tools for binary classification that have been adapted for variable selection. A generalized linear model is broadly defined by

$$\begin{array}{c} g\left(y_{i}\right)=x_{i}^{T}\cdot \beta \end{array}$$

where *y*_*i*_ is the expected value of the random univariate variable *y*_*i*_, *x*_*i*_ is a vector of regressor variables for the *i*th observation, *β* is a vector of parameters or regression coefficients and *g*:(*a*, *b*) → ℝ is a *link* function (usually a sigmoid function such as $g^{-1}(x)=\frac{e^{x}}{1+e^{x}}$ for the *logit* link or *g* ^−1^(*x*) = arctan(*x*) for the *cauchit* link). In the example of microarray data, *x*_*i*_ is a vector of gene expression values for the *i*th microarray sample in an experiment, and *y*_*i*_ is a numeric value corresponding to the experimental condition of the microarray (for example, control condition microarrays might be labeled with *y*_*i*_ = 0, and treatment condition microarrays with *y*_*i*_ = 1). The variable *β* is a vector of free parameters *β*_*j*_, which ‘weights’ the contribution of each gene *j*. The larger (the absolute value of) a component of *β*, the more strongly the corresponding component of *x*_*i*_ contributes to the overall sum $x_{i}^{T}\cdot \beta =\sum_{j=1}^{p}x_{ij}\beta j$, where *p* is the size of *x*_*i*_ (for microarrays, the number of genes on a chip).

Combining all of the samples in an experiment yields the expression

$$\begin{array}{c} g(Y)=X\beta \end{array}$$

where *X* is a matrix of expression values *x*_*ij*_ such that *x*_*ij*_ is the expression value for the *j*th gene from the *i*th sample, *Y* is a vector of classifications of each microarray, and *g*(*Y*) is *g*(⋅) applied to each element of *Y*. The goal of a GLM-based penalized regression technique is to choose *β* such that the above equation holds as closely as possible (i.e., ‖*g*(*Y*) − *Xβ*‖ ≍ 0) while minimizing the number of non-zero components of *β*. The result is a relatively sparse vector *β* whose non-zero components correspond to genes whose expression values contribute meaningfully to successful classification of a sample (in our case, a single microarray), which is taken to indicate differential expression of that gene.

To satisfy the above constraints, our method uses an efficient signal recovery strategy based on a pseudo-likelihood function shown to yield low false discovery rates and high signal recovery relative to other penalized regression methods (for example, Lasso or elastic net) when the number of replications is very small [27]. Our algorithm solves the following optimization problem

$$\begin{array}{c} \left({\hat{\beta }}_{1},{\hat{\beta }}_{2},...{\hat{\beta }}_{p}\right)=\underset{\beta =(\beta_{1},\beta_{2},...\beta_{p})}{\mathrm{argmin}}\{∥\tilde{Y}-\arctan\left(X\beta \right)∥+\lambda \sqrt{∥\beta ∥\cdot {\left|\beta \right|}_{1}}\}, \end{array}$$ (1)

where the components of $\tilde{Y}$ are $\frac{\pi }{2}$ and $-\frac{\pi }{2}$, and *λ* is a constant whose value is determined theoretically [27]. We retain the components of the solution *β* with the highest relative contributions, i.e. $\frac{|\beta_{i}|}{\left\|\beta \right\|}$. The particular choice of penalty term for the penalized Euclidean distance regression enables a unique grouping effect that involves the relative contributions of the components of the solution vector *β*, not just their absolute values. If the angle between columns *i* and *j* (taken as vectors in ℝ^*p*^) of the data matrix *X* is *θ*_*ij*_, then the penalized Euclidean distance regression method produces a vector of weights (rankings) *β* = (*β*_1_, *β*_2_…*β*_*p*_) such that $|\frac{{\hat{\beta }}_{i}(\lambda )}{\left\|\hat{\beta }(\lambda )\right\|}-\frac{{\hat{\beta }}_{j}(\lambda )}{\left\|\hat{\beta }(\lambda )\right\|}|\leq \frac{2\theta_{ij}}{\lambda }.$ Overall, the objective function used by the penalized Euclidean distance method facilitates reconstruction of weak signals in ill-defined situations without pre-estimates of the noise standard deviation. Notably, in numerical simulations with high dimensionality and very weak signals, the false positive rate of PED-based selection was much lower than that of either elastic net or Lasso [27].

Equation (1) can be substituted, and its solution well-approximated, with the computationally simpler problem

$$\begin{array}{c} \left({\hat{\beta }}_{1},{\hat{\beta }}_{2},...{\hat{\beta }}_{p}\right)=\underset{\beta =(\beta_{1},\beta_{2},...\beta_{p})}{\mathrm{argmin}}\{∥\tilde{Y}-X\beta ∥+\lambda \sqrt{∥\beta ∥\cdot {\left|\beta \right|}_{1}}\}, \end{array}$$ (2)

where here the components of $\tilde{Y}$ have large absolute value (here ±10^5^). To simplify computation, our algorithm performs PED regression in two passes. In the first pass, equation (2) is used to select a number of differentially expressed genes as an approximation to the final solution. The genes remaining after the first pass are used to optimize again using equation (1), and all genes with very low weight (∣*β*_*i*_∣ < 10^−6^) are removed.

#### Step III: Numerical Simulations

Once weights (the vector *β*) are assigned to genes, a threshold is chosen to separate differentially expressed and potentially non-differentially expressed genes. Genes with (absolute value) weight larger than the threshold are considered differentially expressed; genes with (absolute value) weight below the threshold are called as non-differentially expressed. Simulations based on the experimental data using a design similar to that of Singhal et al. [39] are used to determine an optimal number of selections. These simulations serve three purposes. Firstly, it “tunes” the threshold parameter, which may need to be set differently for different data sets. Secondly, it provides an estimate of the FDR of selections, which allows for control of the FDR. Finally, it serves as validation of the procedure—in effect, when run, our algorithm tests its own applicability on data resembling the researcher’s.

Simulations were designed with the following constraints:

1. Simulated data should mimic as closely as possible the intensity and differential expression patterns of the real data.
2. Simulated data should share, as much as possible, the correlation structure structure of the real data.
3. It must be known which genes are differentially expressed in simulation and which are not.

Simulations are based on an *n* × *P* matrix of real data *X*, where *X*_*ij*_ is the intensity of *i*th replicate of the *j*th gene. One experimental condition (typically the control condition), is chosen without loss of generality to be represented by the first *k* rows of *M*. The *k* × *P* matrix *X*_*cond*_ consisting of only those replicates is used to generate a simulated data matrix $\tilde{X}$.

To preserve as much correlational structure as possible, the first *k* rows of $\tilde{X}$ are set equal to the first *k* rows of *X*, so that the first experimental condition in simulation is identical to that of the real data. The mean *μ*_*j*_ and standard deviation *σ*_*j*_ of each gene *j* are then estimated from the *j*th column of *X*_*cond*_, and use those estimates to generate Gaussian-distributed data with the same parameters for the second simulation condition. That is, ${\tilde{X}}_{ij}=N(\mu_{j},\sigma_{j})$ when *k* < *i* ≤ *n*.

Differential expression is simulated by multiplying the second condition simulation data by a fold-difference if the fold-difference in the original data is large enough. First, the fold difference *f*_*j*_ in the original data is measured. The fold-difference for a gene *j* is defined as

$$\begin{array}{c} f_{j}=\left\{\begin{array}{cc} \frac{\mu_{j}^{2}}{\mu_{j}^{1}} & :\mu_{j}^{2}\geq \mu_{j}^{1}\\ -\frac{\mu_{j}^{1}}{\mu_{j}^{2}} & \mu_{j}^{2}<\mu_{j}^{1} \end{array}\right. \end{array}$$

where $\mu_{j}^{1}$ is the mean expression value of gene *j* for the first condition and $\mu_{j}^{1}2$ is the mean expression value of gene *j* for the second condition. If ∣*f*_*j*_∣ is greater than or equal to some threshold *T*, then each ${\tilde{X}}_{ij}$ is multiplied by *f*_*j*_ (or by $-\frac{1}{f_{j}}$ if *f*_*j*_ < 0) and that simulated gene is labeled as differentially expressed. If ∣*f*_*j*_∣ < *T*, then the second condition is left unchanged and that simulated gene is labeled as not differentially expressed.

In summary, each simulation data matrix $\tilde{X}$ is defined as:

$$\begin{array}{c} {\tilde{X}}_{ij}=\left\{\begin{array}{cc} X_{ij} & :i\leq k\\ N(\mu_{j},\sigma_{j}) & :i>k,|f_{j}|<T\\ f_{j}*N\left(\mu_{j},\sigma_{j}\right) & :i>k,|f_{j}|\geq T,f_{j}>0\\ -\frac{1}{f_{j}}*N\left(\mu_{j},\sigma_{j}\right) & :i>k,|f_{j}|\geq T,f_{j}<0 \end{array}\right. \end{array}$$

#### Step IV: FDR Estimation and Threshold Tuning

Once several simulations are generated from the user’s data, these simulations are used to estimate the largest number of genes that can be considered as differentially expressed while maintaining the FDR below a threshold (supplied by the user) This is achieved by iteratively increasing the selection size and checking the estimated FDR of the new selection until the FDR increases above the set FDR threshold.

Specifically, PED regression is first performed to rank the genes in each simulation. The FDR is then calculated for a very small selection threshold *n*_*s*0_ by taking the top *n*_*s*0_ genes in each simulation and calculating an empirical FDR, which is simply the number of genes correctly called as differentially expressed in the simulation divided by the selection size. Because the simulations are generated to have similar distributions, levels of signal, and correlation structure to the experimenter’s data, the FDR of selections in simulation is taken as an estimate of the FDR of our real data using the same selection size threshold *n*_*s*0_. The algorithm then iteratively increases the selection size *n*_*s*_ by some Δ*n*_*s*_ until the FDR of any one simulation grows beyond the user-specified threshold value. The last tested *n*_*s*_ before the FDR rises above the FDR threshold becomes the selection size used on the actual data set.

Because the selection of *n*_*s*_ is based on the *maximum* FDR among simulations, we expect selections by this method to be somewhat conservative. However, this choice of criteria for stopping iteration may be sensitive to outliers in FDR. More robust but less conservative stopping criteria could be employed—for instance, iteration could stop when the mean or 90% percentile of FDR among simulations rises above the FDR threshold.

#### Step V: Differential Expression Validation

To additionally guard against false discovery of differential expression when none is actually present, our method employs sample permutation to generate an estimate of the number of selections our method would make in the case of data similar to the user’s, but with no true differential expression. For each data set, the classification vector *Y* for that dataset is randomly permuted, theoretically removing any true differential expression from the data (null signal could also be generated by other methods, such as rotation [40]). Differential expression detection is performed as described above on the permuted data, and the sizes of the selections made are reported.

The result of the differential expression validation is a list of selection sizes made by the algorithm for different permutations of the original data. If there is true differential expression in the dataset, then there should be a strong difference between the number of genes selected by our method in the real data and the number selected in null datasets. In practice, because of the discreteness and limited number of permutations possible at small sample sizes, permutations do not completely destroy correlation between sample label and signal, so that significant numbers of genes can be selected even for permutations. We suggest that if more selections are made in the real data than in any of the permuted data cases, then there is a strong case for true differential expression in the experimenter’s dataset. The farther apart the selection sizes on the real data and permuted data, the greater the strength of evidence for differential expression in the dataset.

An experimenter can quantify the significance of the differential expression validation using Chebyshev’s theorem. Chebyshev’s theorem states that no more than $\frac{1}{k^{2}}$ of the values of any distribution can lie more than *k* standard deviations from the mean. By that rule, for example, a selection size more than 4.5 standard deviations from the mean of the observed null data selection sizes corresponds to a *p*-value of $p<1-\frac{1}{4.5^{2}}=0.05$. Chebyshev’s theorem can be used to estimate a highly conservative *p*-value for finding a selection size as extreme as that of the real data given the empirical distribution of selection sizes in permuted (null) data. This *p*-value is $\frac{1}{k^{2}}$, where *k* is the z-score of the original data selection size when grouped with the selection sizes of the permuted data.

### Method Overview

The following is an algorithmic summary of our selection method.

Input: The user provides a matrix of expression data, as described under “PED regression.” The user also sets an FDR threshold *T*. For instance, for a threshold of *T* = 0.05, no less than 95% of genes selected by the algorithm will actually be differentially expressed.

1. Convert expression data for each gene to z-scores (such that each gene’s expression vector has mean 0 and standard deviation 1) (Step I).
2. Real data first pass: using approximate PED regression according to equation (2), find weights for each gene to define an optimal classifier using the data (Step II).
3. Sort genes by the magnitude of their weights.
4. Generate simulations with known signal based on the real data (Step III).
5. Find a maximum selection size *n*_*s*_ that maintains FDR < *T* using simulations (Step IV):
  (a) For each simulation, set a selection size *n*_*s*_ = *n*_*s*0_.
  (b) Simulation first pass: optimize weights of differentially expressed genes using PED regression according to equation (2) on each simulation. Take the top *n*_*s*_ variables in each simulation as differentially expressed (Step II).
  (c) Simulation second pass: optimize weights of differentially expressed genes using PED regression according to equation (1), then filter out any genes *i* with weight ∣*β*_*i*_∣ < 10^−6^ (Step II).
  (d) Measure the FDR in the selection made in each simulation.
  (e) If the FDR of any simulation’s selection is greater than *T*, stop.
  (f) Otherwise, increment *n*_*s*_ by Δ*n*_*s*_ and go back to 5b.
6. Take the top *n*_*s*_ genes in the real data, sorted by weight according to PED regression.
7. Real data second pass: optimize weights of differentially expressed genes using PED regression according to equation (1), then filter out any genes *i* with ∣*β*_*i*_∣ < 10^−6^ (Step II).
8. Generate permuted versions of the real data as “null signal” cases (9 permutations for *n* = 3; more for larger datasets) (Step V).
9. For each permuted version of the data, perform steps 2–6. Report the number of selections in each permutation and compare to the number of selections in the real data to assess the presence of differential expression (Step V).

### Implementation Details

Code and documentation for PED-based selection are available at https://github.com/sclamons/PED. Gene selection by PED and differential expression validation were implemented as MATLAB scripts, which are also compatible with the free and open-source MATLAB-like environment Octave. The script *PED_select_genes* is used to run our algorithm on a single data set. We also include a script *PED_select_genes_batch* to run our algorithm on multiple datasets with a single command. Null-signal simulations were generated using the script *PED_generate_simulations* with the parameterization *min_fold_diff = inf*. We use the MATLAB package HANSO to solve the optimization problems given in equations (1) and (2).

To simplify computation of the objective function and achieve several theoretical properties during PED regression, we employ the first-pass approximation shown in equation (2), which produces a close approximation of the final solution [27]. Once most genes are filtered out by the first regression and selection, we optimize again with equation (1) and filter out any genes with extremely small weight (< 10^−6^). The results of this second pass are reported as the final selections.

We observed that weighting of genes are somewhat sensitive to the choice of classification vector *Y*, so that the set of genes with the highest weights are not the same when *Y* = [−1, −1, −1, 1, 1, 1]′ as they are when *Y* = [1, 1, 1, −1, −1, −1]′. Thus, for either choice of *Y*, some potentially important genes are missed by PED regression. We therefore perform each optimization twice, once for each version of *Y*, yielding two weights $\beta_{j}^{1}$ and $\beta_{j}^{2}$ for each gene *j*. We then set $\beta_{j}=\max(|\beta_{j}^{1}|,|\beta_{j}^{2}|)$. This way, our algorithm does not lose power due to arbitrary choice of *Y*.

In our implementation, size optimization is performed using 10 simulations per dataset and permutation tests are performed using 9 distinct permutations. To optimize the selection size *n*_*s*_, we first used $n_{s0}=\Delta n_{s}=\frac{n}{1000}$ to roughly estimate the correct choice of *n*_*s*_, then iterated again from the first stopping point with Δ*n*_*s*_ = 1 to more precisely determine optimal selection size.

### Validation

As a negative control experiment, we generated null-signal simulations using the same simulation strategy used in the selection method, but with the fold-difference threshold for differential expression set to +∞ so that no differential expression was introduced. We generated null-signal simulations based on the structure of our Notch-experiment microarray data for each comparison used in that experiment, then applied our selection method to these simulations. This experiment tested the behavior of our method when no differential expression is present in a dataset.

Whole mount in situ hybridization was employed for empirical validation of selected genes. In situ hybridization experiments were carried out using standard published protocols with minor modifications as previously described [30, 41].

Since developmental expression profiles are already known and publicly available for most annotated Xenopus genes on xenbase.org, validation was also performed bioinformatically. Expression information for genes selected as differentially expressed by PED between RNA from GFP injected embryos extracted at st. 18 and RNA extracted from st. 38 was compared with expression profiles for the closely related species *Xenopus tropicalis* available on xenbase.org [42]. GFP injected embryos were selected for this validation because GFP served as a control for the injection procedure and does not affect development.

### Comparison With Other Methods

For comparison, we applied several common penalized regression algorithms to our Notch perturbation dataset. Specifically, we used two implementations of Lasso and Iterative Sure Independence Screening (ISIS) [43]. Lasso was performed with the R package “glmnet”. A fit was calculated using the“cv.glmnet” function with binomial fit family, *α* = 1, and all other parameters default. Bayesian lasso was performed with the R package “monomvn” [44] by using the included function blasso with suggested default values. ISIS was performed with the R package “SIS”, using the function “SIS” with binomial fit family and 3-fold cross validation.

## Results

### Differential Expression Testing With Limma

Microarray data was initially analyzed by the Clemson University Genomics Institute using the limma package in Bioconductor R. We also performed this analysis to confirm the results. Testing for differential expression with limma yielded very few differentially expressed genes (See Table 1).

**Table 1:** Selection sizes for Notch data with Limma.

Stage	DBM v GFP	GFP v NICD
18	1	8
28	0	2
38	0	0

However, an examination of the list of genes with particularly low *p*-values showed that many of the genes with particularly low p values were known through previous molecular studies to be regulated by the Notch signaling pathway. Even though these genes could not be reported as differentially expressed using accepted statistical analysis methods, the presence of so many known Notch regulated genes suggested that this list and the standard approach may be under-representing differentially expressed genes, warranting an alternative method more appropriate for data with low *n* and high dimensionality.

### Differential Expression Testing Using PED

We applied the PED-regression-based method to our microarray data with an FDR threshold of 0.01 in order to recover a more complete list of differentially expressed genes (S1 Table, S2 Table, S3 Table, S4 Table, S5 Table, S6 Table). The results are summarized in Table 2.

**Table 2:** Selection sizes for Notch data and the permutations of the real data with 1% empirical FDR.

	Real Data	Permuted Data									z-score	Chebyshev p-value
18_DBM_18_GFP	781	326	31	36	229	33	34	197	199	322	4.99	0.04
18_GFP_18_NICD	2438	135	29	15	163	27	21	128	118	2149	3.07	0.11
28_DBM_28_GFP	1155	131	40	397	128	163	161	44	70	381	7.40	0.02
28_GFP_28_NICD	1595	56	10	57	60	97	60	17	54	95	52.49	3.6E-4
38_DBM_38_GFP	238	84	99	34	76	54	87	106	83	68	7.24	0.02
38_GFP_38_NICD	752	64	1	0	448	4	3	514	1	0	3.05	0.11

Notably, in every case, our selection method labeled many more genes as differentially expressed in the data than in permuted controls, indicating that these selections are unlikely to be the product of spurious selection of truly random data. All genes that were labeled as differentially expressed by limma (after BHY adjustment) were also selected as differentially expressed by PED.

Selection sizes for our data were consistently greater than selection sizes for null-permuted data. Using Chebyshev’s theorem, we obtained *p*-values for the observed difference in selection sizes. Chebyshev-based *p*-values were only significant for four out of the six comparisons tested. However, it should be noted that because Chebyshev’s theorem does not make any distributional assumptions about the data, it is extremely conservative—it effectively gives an upper bound for *p*-values calculated for *any* assumed distribution.

As a negative control, we generated one simulation with no differential expression for each contrast in our experiment, then applied our selection method to those simulations. The results are summarized in Table 3.

**Table 3:** Selection sizes for simulated data with null signal and its permutations.

	Null Signal Data	Permuted Null Data									z-score	Chebyshev p-value
18_DBM_18_GFP	7	16	14	17	25	30	19	242	198	257	0.782	1
18_GFP_18_NICD	7	11	25	10	26	21	26	196	240	202	0.792	1
28_DBM_28_GFP	0	0	45	14	0	0	0	4	0	0	0.467	1
28_GFP_28_NICD	0	87	23	47	93	55	8	21	27	23	1.41	0.50
38_DBM_38_GFP	8	96	70	69	52	34	49	129	128	118	2.07	0.23
38_GFP_38_NICD	2	39	84	54	48	47	42	43	54	65	3.62	0.08

As a positive control of differential gene expression discovery, we applied our method (again with an FDR threshold set to 0.01) to the comparison: GFP-injected stage 18 versus stage 38. Differential expression in that contrast is driven by transcriptional differences between stages, which are large relative to perturbations induced by DBM or NICD injection. Under these conditions, 20,544 genes were detected as differentially expressed. We obtained similar results by applying limma to the same contrasts with BHY correction at *α* = 0.05 (data not shown).

### Validation of Selection Results

Several different approaches were employed to validate our selection procedure. Firstly, we validated a number of samples empirically. Since the fold differences in our experiments were virtually all significantly less than 2, qRT-PCR was not an appropriate technique, since it reliably detects differences that are more than twofold in magnitude. We therefore conducted in situ hybridization on selected genes and assayed for differences in expression. Of the five genes tested—several of which were not previously known to be regulated by Notch signaling—all five validated the PED selections (data now shown).

Secondly, we validated the selection procedure bioinformatically using existing expression information from multiple databases available on xenbase.org. To do so we compared genes selected as differentially expressed by PED for GFP injected embryos at stage 18 and stage 38 with known expression profiles. GFP was used as an injection control, and GFP embryos display normal development. Of the genes selected as differentially expressed, 200 genes were randomly sampled. Of these 182 (91%) were validated by known expression data from *Xenopus tropicalis*.

Finally, our selection procedure includes a simulation step designed to both validate and tune the procedure for the user’s data set. These simulations use a fold-difference criteria to estimate the level of signal present in the user’s data, then add a random, normally-distributed condition to one of the user’s condition data. Our procedure uses these simulations to tune the selection size to maintain an estimated false discovery rate below a user-set threshold.

### Comparison with Other Methods

A number of analysis methods exist for variable selection using penalized regression techniques. For comparison with our method, we applied lasso, Bayesian lasso, and ISIS, to our dataset. Selection sizes by each method are shown in Table 4. Both methods detected significantly fewer genes as differentially expressed than our method, and in some comparisons detected even less differential expression than Limma.

**Table 4:** Selection sizes for Notch data with other variable selection methods.

Comparison	lasso	Bayesian lasso	ISIS
18_DBM_18_GFP	0	5	1
18_GFP_18_NICD	3	5	1
28_DBM_28_GFP	0	5	1
28_GFP_28_NICD	0	5	1
38_DBM_38_GFP	0	5	1
38_GFP_38_NICD	0	5	1
18_GFP_38_GFP	31	5	1

## Discussion

Although many methods exist for analysis of microarray data, none are known to reliably function for single-channel microarray data with ultra-low sample size, for instance with *n* = 2 or 3. Most statistical tests, such as the *t*-test or even limma, require substantial adjustment for multiple hypothesis testing [5]. This adjustment can be too stringent, leading the investigator to throw out the true positives with the false positives. A large enough sample size can compensate for the low statistical power of adjusted tests, but sample sizes in microarray studies are often limited by cost or sample availability.

Another approach to the analysis of microarray data comes from microarray classification research, which considers the problem of automatically creating a set of rules that can identify the sample type of a previously uncategorized microarray (see Ma and Huang [13] for an overview of classification methods and their application to selection). One challenge for classification algorithms when applied to microarray experiments is the extremely high dimensionality and small number of samples they typically employ. When data is “sparse” in this way, most classification algorithms have no unique solution. Without sufficient constraint, classifiers produce rules describing the noise in the data as well as the underlying biological difference, called “overfitting.”

One solution to the “*p* ≫ *n*” problem is penalized regression, in which solutions to the classification problem are penalized for using additional information [13, 15]. This “soft” form of dimension reduction encourages solutions using a minimum amount of information (taken from a minimum number of variables) over those that overfit, without requiring *a priori* knowledge of the amount or degree of differential expression in the data. Since penalized regression naturally separates significant variables from non-significant variables, the technique can also be used for variable selection, and has been suggested as a means of detecting differentially expressed genes [19, 24]. Unfortunately, classification techniques usually try to extract the *minimum* set of genes required to make a classification, whereas an investigator looking for differential expression in a microarray experiment typically seeks *all* of the differentially expressed genes. Furthermore, the penalized regression methods so far employed for classification and variable selection in microarray experiments typically require tuning using cross-validation [16–23], which is not feasible for experiments with extremely limited sample size [26].

We present a GLM-based, penalized binomial regression approach for analyzing microarray data that uses data-based simulations to tune selections, thus avoiding the need for cross-validation and maximizing the number of differentially expressed genes detected by the algorithm. Because it does not require cross-validation, this method can be applied to experiments with extremely low sample size (*n* > 1), and it can detect large numbers of differentially-expressed genes in cases when exiting methodologies (including lasso, Bayesian lasso, and SIS/ISIS) cannot. Our method has been implemented as a set of functions in MATLAB. As input, the code requires a two-condition experimental matrix in a custom-format CSV file. For ease of use with existing data, we provide a MATLAB script to generate such CSV files from DataMatrix objects (which are produced by many components of MATLAB’s Bioinformatics Toolbox). The code will run on any two-condition experiment with at least two samples per condition. Our algorithm allows the user to choose an acceptable false discovery rate for differential gene discovery. The FDR can be set higher for increased statistical power, or lower for more accurate selections.

We also provide a permutation-based differential expression test, which can verify the presence of differential expression in an otherwise ambiguous dataset. The differential expression test produces selection sizes for sample permutations of the data, which represents a null distribution of selection size. Sets with differential expression will produce much larger selection sizes in the actual data than in the permuted data, while sets with no differential expression will produce similar selection sizes for all tests. We recommend either 1) considering the data differentially expressed if the data show a larger selection size than any permutation or 2) using Chebyshev’s theorem to estimate a highly conservative *p*-value for the selection size, as described in Materials and Methods.

There is potential for expansion of our algorithm. With few modifications, it could be applied to RNA-seq expression data. Our algorithm’s performance is currently quite slow, despite optimization—analysis of a single data set with *n* = 3 and 32,635 genes can take anywhere from hours to a few days on a 4-core Intel machine. Much of the processing time to run our algorithm is devoted to large matrix operations that could be optimized further, delegated to the GPU or other SIMD hardware, or both. Finally, we hope to expand our algorithm to handle complex experimental designs more naturally.

Our method meets an important need for analysis tools capable of analyzing ultra-low sample-size datasets with extremely high dimensionality with enough power to apply pathway analysis and other forms of global expression analysis. Many such datasets exist, and we believe that applying our PED-based approach could yield a plethora of new insights from experiments that have already been performed.

## Supporting Information

S1 Table Differentially expressed genes detected by PED with FDR < 0.01 for Stage 18, DBM vs GFP. (XLS)

S2 Table Differentially expressed genes detected by PED with FDR < 0.01 for Stage 18, GFP vs NICD. (XLS)

S3 Table Differentially expressed genes detected by PED with FDR < 0.01 for Stage 28, DBM vs GFP. (XLS)

S4 Table Differentially expressed genes detected by PED with FDR < 0.01 for Stage 28, GFP vs NICD. (XLS)

S5 Table Differentially expressed genes detected by PED with FDR < 0.01 for Stage 38, DBM vs GFP. (XLS)

S6 Table Differentially expressed genes detected by PED with FDR < 0.01 for Stage 38, GFP vs NICD. (XLS)
